# Past trends and future projections of palliative care needs in Chile: analysis of routinely available death registry and population data

**DOI:** 10.1186/s12916-024-03570-1

**Published:** 2024-09-02

**Authors:** Javiera Leniz, Angélica Domínguez, Anna E. Bone, Simon Etkind, Pedro E. Perez-Cruz, Katherine E. Sleeman

**Affiliations:** 1 Departamento de Salud Pública, Escuela de Medicina, Pontificia Universidad Católica de Chile, Santiago, Chile; 2 Cicely Saunders Institute of Palliative Care, Policy and Rehabilitation, King’s College London, London, UK; 3 Department of Public Health and Primary Care, University of Cambridge, Cambridge, UK; 4 Cambridge University Hospitals NHS Foundation Trust, Cambridge, UK; 5 Sección Medicina Paliativa, Escuela de Medicina, Pontificia Universidad Católica de Chile, Santiago, Chile

**Keywords:** Palliative care needs, Projections, Population estimations, Serious health-related suffering

## Abstract

**Background:**

The number of people with palliative care needs is projected to increase globally. Chile has recently introduced legislation for universal access to palliative care services for patients with severe and terminal illnesses, including non-cancer conditions. We aimed to estimate the number of people affected by serious health-related suffering and need for palliative care in Chile to 2050.

**Methods:**

We used data on all deaths registered in Chile between 1997-2019 and population estimates for 1997–2050. We used Poisson regression to model past trends in causes of death adjusted by age, sex and population estimates, to project the number of deaths for each cause from 2021 to 2050. We applied the Lancet Commission on Palliative Care and Pain Relief weights to these projections to identify decedents and non-decedents with palliative care needs.

**Results:**

Population palliative care needs in Chile are projected to increase from 117 (95% CI 114 to 120) thousand people in 2021 to 209 (95% CI 198 to 223) thousand people in 2050, a 79% increase (IRR 1.79; 95% CI 1.78–1.80). This increase will be driven by non-cancer conditions, particularly dementia (IRR 2.9, 95% CI 2.85–2.95) and cardiovascular conditions (IRR 1.86, 95% CI 1.83–1.89). By 2050, 50% of those estimated to need palliative care will be non-decedents (not expected to die within a year).

**Conclusions:**

Chile will experience a large increase in palliative care needs, particularly for people with dementia and other non-cancer conditions. Improved availability of high-quality services, expanded clinician training and new sustainable models of care are urgently required to ensure universal access to palliative care.

**Supplementary Information:**

The online version contains supplementary material available at 10.1186/s12916-024-03570-1.

## Key messages

- Estimating the number of people who experience serious health-related suffering and might benefit from palliative care need is key for service planning. This is particularly relevant in Chile, where a new law has been implemented that guarantees access to palliative care services for all patients with terminal conditions and severe diseases.
- We estimated the number of people who will experience serious health-related suffering and palliative care needs in Chile by 2050.
- The number of people with palliative care needs in Chile is going to almost double by 2050, mainly due to an increase in the number of people living and dying with dementia and non-cancer conditions.
- The projected increase in the number of people with palliative care needs highlights the need for enhancing availability of services, increase personnel training and new sustainable models of care, in particular for non-cancer conditions.

**Supplementary Information:**

The online version contains supplementary material available at 10.1186/s12916-024-03570-1.

## Background

Globally, it is estimated that around 25 million people die every year with serious health-related suffering. This global burden is projected to almost double by 2060, with the greatest increase in low- and middle-income countries [1]. Most of this increase is driven by increases in the number of people dying from cancer, cardiovascular disease and dementia [1]. People living with serious illnesses can experience physical and psychological symptoms that have an impact not only on the patient but also on caregivers and society [2–5]. There is evidence pointing towards cost-effectiveness of some palliative care service models [6], and palliative care has been included as a key aspect of Universal Healthcare Access [7]. Given the projected increase in serious health-related suffering, universal access to palliative care services has become a public health priority.

According to the Global Atlas of Palliative Care, most countries in Latin America are in an early stage of palliative care service development, with low access to palliative care services [8]. Chile implemented a pain management program for cancer patients in 1995, which was later enhanced by a more comprehensive palliative care service for people with advanced cancer under the Health Explicit Guarantee’s Law in 2004 [9]. These schemes have contributed to an increase in access to opioids and palliative care services for people with cancer in Chile [10]. In 2019, around 44,000 cancer patients accessed palliative care services, representing 93% of the total number of cancer patients in need of palliative care [11], but only 42% of patients (including both patients with cancer and non-cancer conditions) in need of palliative care accessed palliative care services [11]. Regardless of this gap in access, Chile was ranked among the countries with the best access to palliative care in Latin America [10], and 27th in the 2015 Quality of Death Global Index [12].

In 2022, a new law that guarantees access to palliative care services for all patients with terminal conditions and severe diseases was introduced in Chile [13], enforcing the need to expand services to provide palliative care access to all those who need it. Two studies have investigated the demand for palliative care services in Chile between 2018 and 2020 [11, 13]. Using the Lancet Commission method to estimate population palliative care needs, Perez-Cruz et al. estimated that 104,923 patients would have benefited from palliative care in 2019 in Chile. Fifty-six percent of them were affected by non-cancer conditions and only 51% were in the last year of life [11]. Using different methodologies, Armijo et al. estimated that between 2018 and 2020, 21,679–25,650 people with non-cancer conditions needed palliative care in the last year of life each year [13].

While access to palliative care services among cancer patients in Chile is relatively high, this does not necessarily imply high-quality care or a regular provision of services. To support effective healthcare planning, it is necessary to understand both current and future needs. We aimed to estimate the number of people affected by serious health-related suffering and need for palliative care in Chile by 2050, to support a planned increase in high-quality palliative care services.

## Methods

### Design

Secondary analysis of routinely available national death registry and population estimates data provided by the Department of Health Information and Statistics (DEIS) and the Institute for National Statistics (INE) in Chile respectively. We used death registry data and population estimates to identify past trends in the number of deaths by cause of death and then to derive future trends. We used population estimates to account for the demographic structure of the population.

### Data sources

#### Death registry data

Death registry data in Chile is collected, verified and published jointly by the INE, Civil Register Office and the DEIS [14]. Certification of death by a physician is mandated by law and the coverage and quality of death certificates have been judged as high [14, 15]. Death certificates use the standard World Health Organization International form which records the sequence of diseases that led directly to death and all other contributing causes of death [16]. Based on the information recorded on death certificates, trained staff from the DEIS manually select and code the underlying cause of death based on the International Statistical Classification of Diseases and Related Health Problems 10th revision (ICD-10) guidelines [17, 18]. A death registry dataset that contains all deaths registered in the country between 1990 and 2020, except external causes of death, is publicly available from the DEIS website [19]. We extracted individual-level information on age, sex, year of death and underlying cause of death for all deaths registered in Chile between 1997 and 2019 from that dataset [19]. Pre-1997 deaths were excluded due to changes in how cause of death was coded. We excluded deaths during 2020 because temporary changes in cause of deaths from the COVID-19 pandemic would have skewed the projection model.

#### Population estimates

Population estimates in Chile are regularly produced by the INE. These estimates contain information on the population size and demographic structure by year and are based on information from the last census and a set of assumptions on fertility, mortality and international migration trends [20]. A dataset with these population estimates is available from the INE website [20]. We extracted information on the estimated population in Chile between 1997 and 2050, stratified by age and sex from the most recent population estimation and projections for Chile, which was based on the 2017 census [20].

### Palliative care needs estimations

To estimate the number of people who might need palliative care, we used the method developed by the Lancet Commission on Global Access to Palliative Care and Pain Relief [21]. We used this approach as it has been used in previous research exploring palliative care needs in Chile and elsewhere [1, 11, 21]. Based on expert opinion, the Lancet Commission identified a set of 20 conditions that most commonly result in death or in suffering that is severe enough to benefit from palliative care, including conditions that affect children [21]. Estimates of palliative care needs are then derived based on the number of people who die from these 20 conditions, separately for decedents and non-decedents. The Lancet Commission provided methods to estimate the proportion of decedents with each condition who need palliative care during their last year of life, based on symptom prevalence. In addition, because palliative care might be expected to benefit people with some conditions before their last year of life, the Lancet Commission provided methods to estimate the number of non-decedents who need palliative care for 11 relevant conditions (expressed relative to the number of decedents for each condition in the population). Table 1 shows the 20 conditions identified by the Lancet Commission and the ICD-10 related codes we used. Originally, the Lancet Commission codes for dementia only included ICD-10 codes F00–F04, while ICD-10 codes “G30: Alzheimer disease” and “G31.1: Senile degeneration of brain” which are commonly used to identify people who died from dementia [22–24] were included in the section “Central nervous system (CNS) non-inflammatory conditions”. As G30 and G31 codes are commonly used in Chile to report dementia deaths, we used them to identify dementia deaths rather than other CNS degenerative conditions [22–24]. We calculated the annual number of decedents (for the 20 conditions) and non-decedents (for the 11 conditions) between 1997 and 2019 and applied the weights defined by the Lancet Commission to estimate the number of decedents and non-decedents with palliative care needs (1997–2019) [21].

**Table 1:** The 20 conditions most likely to cause serious health-related suffering that requires palliative care, as identified in the Lancet Commission on Global Access to Palliative Care and Pain Relief, and the ICD-10 codes used [21]

Condition category	Conditions	ICD-10 code
Cancer	Malignant neoplasm, excluding leukaemia*	C00–C97 (except C91–C95)
	Leukaemia	C91–C95
Cardiovascular diseases	Cerebrovascular disease*	I60–I69
	Heart rheumatic disease, cardiomyopathies and heart failure	I05–I09, I10–I15, I42, I50
	Ischemic heart disease	I25
Chronic organ failure	Chronic lung disease	J40–47, J60–70, J80–84, J95–99
	Liver disease	K70–K77
	Chronic renal kidney	N17–N19
	Musculoskeletal diseases*	M00–M97
	Atherosclerosis (not included in other categories)	I70
Communicable diseases	Haemorrhagic fevers*	B33.4
	TB*	A15–A19
	HIV*	B20–B24
	CNS inflammatory conditions*	G00–G09
Dementia	Dementia*	F00–F04, G30–G32
Other	CNS non-inflammatory conditions*	G20–G26, G35–G37, G40–G41, G80–83
	Preterm birth complications and birth trauma	P07, P10–P15
	Congenital malformations*	Q00–Q99
	Wounds, intoxications, external causes*	S00–S99, T00–T98, V01–Y98
	Malnutrition	E40–E46

*Conditions where palliative care needs were estimated for decedents and non-decedents

*CNS* Central Nervous System, *TB* Tuberculosis, *HIV* Human Immunodeficiency Virus

### Death projections

To project palliative care needs, first we calculated the number of deaths for each of the 20 conditions likely to cause serious health-related suffering and need for palliative care, by category of age (by 5-year intervals), sex (male, female) and year of death (1997–2019). As 2020 was an anomalous year, with an unusually high number of deaths caused by COVID–19 infection, we did not include deaths in 2020. As the number of deaths by year was highly variable for less prevalent conditions, we used a simple moving average data smoothing approach, calculating the average of the number of deaths from previous, current and following year for each condition. For deaths in 1997 and 2019, a two-point average (current and nearest year) was used [25].

We used Poisson regression models to calculate the estimated number of deaths for each of the 20 conditions by year (1997–2019), adjusted by age, sex and the exposed population. These models allowed us to estimate past trends and to then predict the age and sex adjusted number of deaths (with 95% confidence intervals (CI)) for 2020–2050 using the forecast function in Stata and based on the INE population projections.

Finally, we applied the weights from the Lancet Commission method to the estimated past deaths and projected deaths for each of the 20 conditions, adjusted by age and sex, to estimate the number of decedents and non-decedents likely to have serious health-related suffering and need for palliative care from 2020 to 2050. The 20 conditions were grouped into cancer, cardiovascular diseases, chronic organ failure, non-communicable diseases, dementia and other conditions (Table 1). More information on the weights used is available in Additional file 1: Table S1. More detail in the methods followed is available in Additional file 2. The Stata® 17.0 software was used for the analysis.

## Results

Between 1997 and 2019, the annual number of deaths in Chile rose from 78,472 to 109,658, with a mortality rate from 5.3 to 5.7 per 1000 people. In 1997, based on the Lancet Commission weightings, 34,957 (44.5%) of the 78,472 observed deaths were from conditions likely to require palliative care. This proportion increased to 52,645 (48.0%) in 2019 (Additional file 1: Table S2).

The number of decedents with palliative care needs is estimated to increase from 57,596 (95% CI 56,325 to 58,953) in 2021 to 104,809 (95% CI 99,746 to 111,612) in 2050 (Table 2 and Additional file 1: Table S3). This represents an 82% increase in the number of decedents with palliative care needs (incidence risk ratio (IRR) 1.82, 95% CI 1.80 to 1.84) between 2021 and 2050. The number of people with palliative care needs who are not expected to die within a year (non-decedents) is expected to increase from 58,869 (95% CI 57,244 to 60,634) in 2021 to 103,688 (95% CI 97,907 to 111,379) in 2050 representing a 76% increase (IRR 1.76; 95% CI 1.74–1.78). Overall, the number of people with palliative care needs who are not expected to die within a year represents 50% of the total number of people with palliative care needs (Table 2 and Additional file 1: Table S3).

**Table 2:** Estimated number of decedents and non-decedents with palliative care needs in Chile in 2021 and 2050

	**2021**	**2050**	**IRR (95% CI)**
	**n (95% IC)**	**n (95% IC)**	
Decedents	57,595.8 (56,324.5–58,952.5)	104,808.5 (99,746.1–111,611.5)	1.82 (1.80–1.84)
Non-decedents	58,868.6 (57,243.6–60,634.0)	103,687.9 (97,907.1–111,378.6)	1.76 (1.74–1.78)
Total	116,464.4 (113,568.1–119,586.5)	208,496.4 (197,653.1–222,990.1)	1.79 (1.78–1.80)

**Fig. 1:**
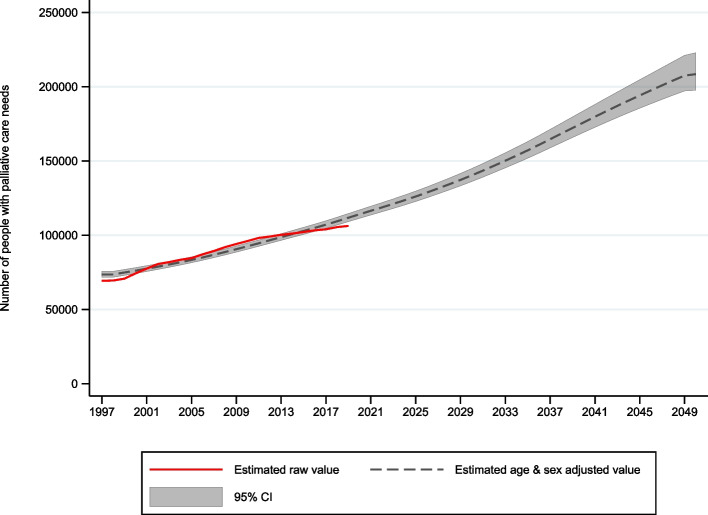
Estimated total (decedents and non-decedents) number of people with palliative care needs in Chile 1997-2050

Including the number of people who might benefit from palliative care before the last year of life (non-decedents), the total estimated number of people with palliative care needs is projected to increase from 116,464 (95% CI 113,568 to 119,587) in 2021 to 208,496 (95% CI 197,653 to 222,990) in 2050 (Fig. 1), a 79% increase (IRR 1.79, 95% CI 1.78 to 1.80).

### Palliative care needs by condition

Dementia and cardiovascular diseases are the conditions with the highest expected increase in the number of people with palliative care needs. The number of people with palliative care needs from dementia is expected to increase from 17,851 (95% CI 17,449 to 18,271) in 2021 to 51,828 (95% CI 49,312 to 54,478) in 2050, representing a 190% increase (Table 3). The number of people with palliative care needs from cardiovascular diseases is projected to increase from 17,038 (95% CI 16,706 to 17,380) in 2021 to 31,689 (95% CI 30,441 to 32,992) by 2050, representing an 86% increase (Table 3).

**Table 3:** Predicted number of decedents and non-decedents with palliative care needs in Chile in 2021 and 2050 by group of conditions

	2021	2050	IRR (95% CI)*
	*N* (95% CI)	*N* (95% CI)	
Cancer			
Decedents	26,086.5 (25,755.9–26,425.3)	39,521.1 (38,636.3–40,438.9)	1.52 (1.49–1.54)
Non-decedents	23,790.2 (23,530.3–24,053.8)	35,904.3 (35,209.4–36,613.3)	1.51 (1.48–1.53)
Total	49,876.7 (49,286.2–50,479.1)	75,425.4 (73,845.8–77,052.2)	1.51 (1.50–1.53)
Cardiovascular diseases			
Decedents	11,673.1 (11,431.6–11,923.3)	26,242.0 (25,161.9–27,371.5)	2.25 (2.20–2.30)
Non-decedents	5364.4 (5274.1–5456.8)	5447.2 (5279.4–5620.4)	1.02 (0.98–1.05)
Total	17,037.5 (16,705.7–17,380.1)	31,689.2 (30,441.3–32,991.9)	1.86 (1.83–1.89)
Chronic organ failure			
Decedents	10,714.9 (10,390.5–11,054.7)	18,620.3 (17,554.4–19,758.7)	1.74 (1.70–1.78)
Non-decedents	980.7 (910.5–1057.5)	1774.1 (1561.3–2016.4)	1.81 (1.67–1.96)
Total	11,695.6 (11,301.0–12,112.2)	20,394.3 (19,115.7–21,775.0)	1.74 (1.70–1.78)
Dementia			
Decedents	3889.1 (3801.5–3980.5)	11,291.5 (10,743.3–11,868.9)	2.90 (2.80–3.01)
Non-decedents	13,961.8 (13,647.6–14,290.1)	40,536.4 (38,568.4–42,609.4)	2.90 (2.85–2.96)
Total	17,850.8 (17,449.1–18,270.6)	51,827.8 (49,311.7–54,478.3)	2.90 (2.85–2.95)
Communicable diseases			
Decedents	786.0 (701.9–896.9)	1462.3 (761.5–3593.2)	1.86 (1.71–2.03)
Non-decedents	7785.9 (7174.6–8480.8)	7360.0 (5857.1–10,421.1)	0.95 (0.92–0.98)
Total	8572.0 (7876.5–9377.8)	8822.3 (6618.6–14,014.3)	1.03 (1.00–1.06)
Other			
Decedents	4446.2 (4243.1–4671.8)	7671.4 (6888.6–8580.4)	1.73 (1.66–1.79)
Non-decedents	6985.6 (6706.5–7295.0)	12,666.0 (11,431.4–14,098.0)	1.81 (1.76–1.87)
Total	11,431.8 (10,949.6–11,966.8)	20,337.4 (18,320.0–22,678.4)	1.78 (1.74–1.82)

*IRR of predicted numbers in 2050 versus 2021

In 2050, the estimated number of people with palliative care needs from cancer is 75,425 (95% CI 73,846 to 77,052). Both, decedents and non-decedents with palliative care needs from cancer conditions are expected to increase 51% between 2021 and 2050. People with palliative care needs from chronic organ failure and communicable diseases are expected to increase by 74% (IRR 1.74, 95% CI 1.70 to 1.78) and 3% (IRR 1.03, 95% CI 1.00 to 1.06) respectively (Table 3).

**Fig. 2:**
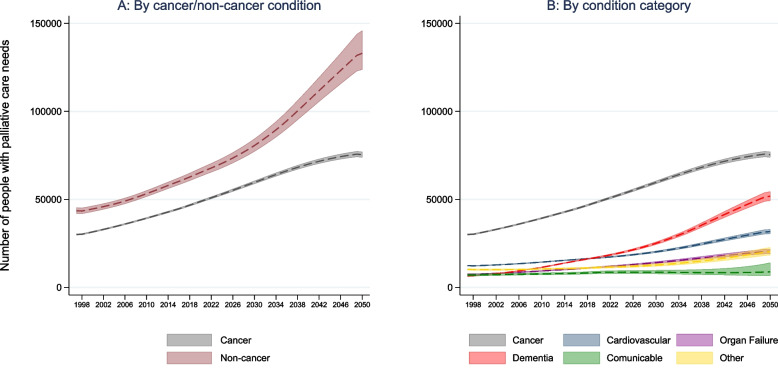
Estimated total (decedents and non-decedents) number of people with palliative care needs in Chile by cancer/non-cancer condition and by condition category

Overall, the number of people with palliative care needs from non-cancer conditions will be almost double the number of people with palliative care needs from cancer conditions by 2050 (Fig. 2).

## Discussion

Results from our analysis suggest the number of people with palliative care needs in Chile is going to almost double by 2050. In absolute terms that means between 197,653 and 222,990 people might require palliative care by 2050. The number of people with palliative care needs from non-cancer conditions is higher, and is growing faster, than those with cancer, mainly explained by the projected increase in the number of people living and dying with dementia and cardiovascular conditions.

Our results suggest Chile is going to experience a significant increase in the number of people with palliative care needs. This highlights the importance of expanding high-quality service provision for the Chilean population. These results align with global projections for upper-middle income countries using the Lancet Commission method [1]. Studies from other high-income countries such as England, Wales and Scotland have estimated smaller increases in the number of people requiring palliative care (between a 25.4% and a 43%) [26, 27]. In Latin America, dos Santos et al. estimated a 76% increase in the number of people with palliative care needs by 2040 in Brazil, similar to our estimations [28].

These projections are likely to be a reflection of the increase in life expectancy and reduction in age-standardised mortality rate, observed and projected for most leading causes of death (including cardiovascular disease and dementia) in Chile and other Latin American countries [29–31]. This suggests population growth and ageing is driving the projected increase in palliative care needs. Our estimations suggest that by 2050 there will be around 130 thousand people in need of palliative care for non-cancer conditions in Chile, almost double the number of people with cancer conditions. Until now, service provision in Chile has been organised to provide palliative care mainly for cancer patients [11]. While the introduction of the new law that guarantees the right to access palliative care services for all patients with terminal conditions in Chile including non-cancer conditions is welcome, there will need to be a large expansion of palliative care services to meet the need. A previous study estimated that the cost of providing palliative care in Chile is on average $4606 United States dollars (USD) per person for cancer and $2285 USD per person for non-cancer conditions [11]. Our findings indicate that providing palliative care to comply with the new law will require a large economic investment: assuming the cost per person remains the same, the cost of providing care for cancer and non-cancer conditions in 2050 will be approximately double the cost of providing palliative care for only cancer conditions and three times larger than the cost of providing palliative care for only cancer patients in 2020.

These findings stress the importance of expanding high-quality palliative care services for both cancer and non-cancer conditions. According to the Latin American Atlas of Palliative Care, Chile had 244 palliative care services in 2018, representing 13.4 palliative care services per million people [32]. A rise in specialist palliative care units across the country and new models of palliative care needs optimised for non-cancer conditions are needed. Models of care that integrate palliative care and primary care teams have been shown to reduce symptom burden, reduce hospital care and have been cost-effective in non-cancer conditions and older adults [33–35]. This requires increasing palliative care training among healthcare professionals both at undergraduate and postgraduate levels and enhancing the provision of palliative care in primary care through more training and resources [36, 37]. However, to secure access to high-quality palliative care for all patients in need, increased coverage should be jointly addressed with strategies for evaluating and improving the quality of care provided [38].

Based on past trends and population estimations, our results suggest the number of people living and dying with dementia who experience palliative care needs will increase threefold by 2050. These findings are consistent with global projections that suggest dementia is the condition with the highest proportional increase in serious-related suffering [1]. Moreover, it is likely the burden of dementia is underestimated in our analysis due to under recognition of dementia as an underlying or contributing cause of death in death certificates [39]. Most people with dementia experiencing palliative care needs are people who will not die in the following year and might require palliative care for several years. Understanding how to provide good quality of end-of-life care for people with dementia in this context is key. Researchers are currently exploring models of palliative care for people with dementia in Australia, the United States of America (USA) and Europe [40–44]. However, there is an urgent need to understand how these models or interventions might be translated to the Chilean context.

### Strengths and limitations

This study has several strengths. We used data on all deaths in Chile, adjusted by age and sex, and modelled future projections based on past trends using more than 20 years of data, as well as including changes in population estimates. We used the Lancet Commission methods previously used for global estimations, and also in the Chilean context, which facilitates comparisons. We also included estimations of palliative care needs for non-decedents, which is important to provide a more realistic estimation of service demand.

Estimating palliative care needs from death certificates records has limitations, as it relies on the quality of records and is sensitive to changes in medical certification and coding practices. The Lancet Commission weightings are based on international expert consensus and might not be representative of Chilean symptom prevalence and care needs. Non-decedents are estimated from decedent cases which do not necessarily represent prevalent cases. Using a Poisson model allowed us to project future trends from past trends while adjusting by age and sex and considering population estimates. However linear estimations assume past trends will remain stable which is not necessarily true. Changes in service provision, disease prevalence and incidence, disease trajectories and treatment, as well as new emergent diseases such as COVID-19 might affect these trends, and therefore long-term projections should be interpreted cautiously.

## Conclusions

The number of people with palliative care needs in Chile is going to almost double by 2050. Palliative care needs among people with non-cancer conditions are growing faster than those with cancer, mainly explained by the projected increase in the number of people living and dying with dementia and cardiovascular conditions. Improved availability of services, palliative care training and new sustainable models of care are urgently required to ensure universal access to palliative care for those who need it.

### Supplementary Information

Additional file 1: Past Trends and future projections of palliative care needs in Chile: analysis of routinely available death registry and population data.

Additional file 2: Methodology supplement.

## Data Availability

Mortality data used in this study is available from the Department of Health Information and Statistics (DEIS) website (https://deis.minsal.cl/). Population estimates used in this study are available from the Institute for National Statistics (INE) website (https://www.ine.gob.cl/). Specific codes and weights used to derive palliative care needs and Stata codes used in the analysis are available in Additional file 1. The last version of the data used for graphs and tables is available at: Leniz Martelli, Javiera. Conjunto de datos: Past trends and future projections of palliative care needs in Chile: analysis of routinely available death registry and population data. Datos de Investigación UC https://doi.org/10.60525/04teye511/T44IRY. V1 (2024)

## Abbreviations

DEIS: Department of Health Information and Statistics
INE: Institute for National Statistics
ICD-10: International Statistical Classification of Diseases and Related Health Problems 10th revision
CNS: Central nervous system
IRR: Incidence risk ratio
CI: Confidence interval
USD: United States dollars
USA: United States of America
