# Supplementary material for: Past trends and future projections of palliative care needs in Chile: analysis of routinely available death registry and population data
Source: BMC Med. 2024 Sep 2;22:350. doi: 10.1186/s12916-024-03570-1 (PMC11367822; doi:10.1186/s12916-024-03570-1)
Supplement: Supplementary file 1 — Additional file 1: Past Trends and future projections of palliative care needs in Chile: analysis of routinely available death registry and population data. [file 12916_2024_3570_MOESM1_ESM.pdf]

Additional file 1: Past Trends and future projections of palliative care needs in Chile: analysis of routinely available death registry and population data

Table S1: Lancet Commission on Global Access to Palliative Care and Pain Relief weights 2

Table S2: Number of deaths and mortality rate in Chile between 1997-2019 and the estimated number of decedents with palliative care needs... 3

Table S3: Predicted number of decedents and non-decedents with palliative care needs in Chile between 1997-2050.. 4

Table S1: Lancet Commission on Global Access to Palliative Care and Pain Relief weights

Condition category	Conditions	CIE-10 code	PC needs factor for decedents	Factor for non-decedents(1)
Cancer	Malignant neoplasm, excluding leukemia	C00-C97 (except C91-C95)	90%	0.94
	Leukemia	C91-C95	90%	
Cardiovascular diseases	Cerebrovascular disease	I60-I69	65%	0.95
	Heart rheumatic disease, cardiomyopathies, and heart failure	I05-I09, I10-I15, I42, I50	Rheumatic fever (65%); Heart hypertensive disease (70%); cardiomyopathy, myocarditis endocarditis (40%); Chagas disease (30%)	
	Ischemic heart disease	I25	5%	
Comunicable diseases	Hemorrhagic fevers	B33.4	100%	1.00
	TB	A15-A19	100%	0.08
	HIV	B20-B24	100%	14.87S
	CNS inflammatory conditions	G00-G09	Syphilis (G01 - 70%); Measles (G02 - 50%); Meningitis (G00, G03 - 30%); Enkefalitis (G04, G05 - 30%); Tripasonomiasis (G028 - 100%)	0.03
Dementia	Dementia	F00-F04, G30-G32	80%	3.59
Other	CNS non-inflammatory conditions	G20-G26, G35-G37, G40-G41, G80-83	Parkinson's disease (G20-G26 - 65%); Epilepsy (G40-G41 - 50%); Multiple sclerosis (G35-G37 - 100%); Other neurological conditions (G30-G32, G80-83 - 65%)	1.67
	Chronic lung disease	J40-47, J60-70, J80-84, J95-99	Chronic obstructive lung disease (J40-J47 - 80%); Other (J60-J70, J80-J84, J95-J99 - 50%)	
	Liver disease	K70-K77	95%	
	Chronic renal kidney	N17-N19	45%	
	Preterm birth complications and birth trauma	P07, P10-P15	Preterm birth complications (P07 - 75%); Birth asphyxia and birth trauma (P10-P15 - 40%)	
	Congenital malformations	Q00-Q99	60%	1.00
	Wounds, intoxications, external causes	S00-S99, T00-T98, V01-Y98	30%	2.00
	Atherosclerosis (not included in other categories)	I70	35%	
	Musculoskeletal diseases	M00-M97	70%	2.00
	Malnutrition	E40-E46	100%	

(1) Factor applied to the number of people with palliative care needs from decedents.

Table S2: Number of deaths and mortality rate in Chile between 1997-2019 and the estimated number of decedents with palliative care needs

Year	Estimated population (thousand people)	Total number of deaths n	Estimated decedents with palliative care needs n	%	Mortality rate (x1000)
1997	14,783	78,472	34,957	44.5	5.31
1998	14,975	80,257	35,313	44.0	5.36
1999	15,162	81,984	36,101	44.0	5.41
2000	15,343	78,814	37,176	47.2	5.14
2001	15,520	81,871	38,202	46.7	5.28
2002	15,692	81,079	39,548	48.8	5.17
2003	15,857	83,672	40,462	48.4	5.28
2004	16,022	86,138	41,365	48.0	5.38
2005	16,183	86,102	41,864	48.6	5.32
2006	16,348	85,639	42,989	50.2	5.24
2007	16,518	93,000	43,960	47.3	5.63
2008	16,698	90,168	45,223	50.2	5.40
2009	16,881	91,965	46,327	50.4	5.45
2010	17,064	97,930	47,252	48.3	5.74
2011	17,254	94,985	48,320	50.9	5.51
2012	17,443	98,711	48,666	49.3	5.66
2013	17,612	99,770	49,488	49.6	5.66
2014	17,788	101,960	50,170	49.2	5.73
2015	17,971	103,327	50,870	49.2	5.75
2016	18,167	104,026	51,615	49.6	5.73
2017	18,419	106,388	52,009	48.9	5.78
2018	18,751	106,796	52,498	49.2	5.70
2019	19,107	109,658	52,645	48.0	5.74

Table S3: Predicted number of decedents and non-decedents with palliative care needs in Chile between 1997-2050.

Year	Predicted number of decedents with palliative care needs<br><br>N (95% CI)	Predicted number of non-decedents with palliative care needs<br><br>N (95% CI)	Predicted number of people with palliative care needs<br><br>N (95% CI)
1997	36,770.2 (35,850.3 - 37,758.1)	36,859.7 (35,772.0 - 38,040.7)	73,629.9 (71,622.3 - 75,798.8)
1998	36,712.9 (35,816.2 - 37,674.9)	36,922.4 (35,852.0 - 38,084.4)	73,635.3 (71,668.2 - 75,759.3)
1999	37,288.8 (36,401.0 - 38,240.1)	37,575.9 (36,508.2 - 38,734.3)	74,864.7 (72,909.2 - 76,974.5)
2000	37,892.4 (37,011.8 - 38,834.9)	38,249.4 (37,183.5 - 39,405.5)	76,141.8 (74,195.3 - 78,240.4)
2001	38,523.7 (37,648.8 - 39,459.2)	38,943.5 (37,878.2 - 40,098.5)	77,467.2 (75,526.9 - 79,557.8)
2002	39,185.4 (38,314.4 - 40,115.9)	39,662.2 (38,596.0 - 40,817.6)	78,847.6 (76,910.4 - 80,933.5)
2003	39,879.7 (39,010.7 - 40,807.1)	40,410.2 (39,341.5 - 41,567.9)	80,289.8 (78,352.3 - 82,375.0)
2004	40,608.8 (39,740.0 - 41,535.4)	41,190.6 (40,117.6 - 42,352.7)	81,799.4 (79,857.5 - 83,888.0)
2005	41,381.8 (40,510.7 - 42,310.0)	42,010.9 (40,931.3 - 43,179.9)	83,392.7 (81,442.0 - 85,489.9)
2006	42,200.0 (41,324.3 - 43,132.6)	42,871.8 (41,783.0 - 44,050.5)	85,071.8 (83,107.2 - 87,183.1)
2007	43,066.7 (42,183.6 - 44,006.5)	43,777.7 (42,676.8 - 44,969.3)	86,844.4 (84,860.5 - 88,975.9)
2008	43,972.6 (43,079.7 - 44,922.5)	44,722.7 (43,606.9 - 45,930.3)	88,695.3 (86,686.6 - 90,852.8)
2009	44,907.9 (44,002.9 - 45,870.4)	45,698.8 (44,565.4 - 46,925.5)	90,606.8 (88,568.3 - 92,795.9)
2010	45,871.5 (44,952.0 - 46,849.0)	46,705.5 (45,551.5 - 47,954.4)	92,577.0 (90,503.6 - 94,803.4)
2011	46,860.1 (45,923.8 - 47,855.3)	47,738.3 (46,560.9 - 49,012.5)	94,598.4 (92,484.7 - 96,867.7)
2012	47,866.6 (46,911.0 - 48,882.0)	48,787.8 (47,584.0 - 50,090.5)	96,654.3 (94,495.0 - 98,972.4)
2013	48,880.3 (47,903.0 - 49,918.7)	49,843.0 (48,609.8 - 51,177.6)	98,723.3 (96,512.8 - 101,096.3)
2014	49,896.6 (48,894.7 - 50,961.1)	50,896.5 (49,630.5 - 52,266.7)	100,793.0 (98,525.2 - 103,227.8)
2015	50,914.3 (49,885.1 - 52,008.0)	51,948.7 (50,646.3 - 53,358.6)	102,863.0 (100,531.4 - 105,366.7)
2016	51,936.9 (50,877.3 - 53,063.3)	53,010.3 (51,667.1 - 54,464.7)	104,947.2 (102,544.4 - 107,527.9)
2017	53,006.9 (51,912.7 - 54,170.4)	54,127.8 (52,737.9 - 55,633.3)	107,134.7 (104,650.6 - 109,803.7)
2018	54,119.5 (52,986.3 - 55,325.2)	55,299.4 (53,856.4 - 56,863.1)	109,418.9 (106,842.8 - 112,188.3)
2019	55,278.2 (54,101.6 - 56,531.2)	56,516.3 (55,014.6 - 58,144.9)	111,794.5 (109,116.2 - 114,676.1)
2020	56,434.0 (55,211.2 - 57,737.4)	57,707.5 (56,144.7 - 59,403.8)	114,141.5 (111,355.9 - 117,141.2)
2021	57,595.8 (56,324.5 - 58,952.5)	58,868.6 (57,243.6 - 60,634.0)	116,464.4 (113,568.1 - 119,586.5)
2022	58,768.4 (57,446.1 - 60,181.1)	60,005.2 (58,317.2 - 61,841.2)	118,773.6 (115,763.4 - 122,022.3)
2023	59,969.3 (58,592.8 - 61,442.0)	61,153.2 (59,399.5 - 63,063.0)	121,122.4 (117,992.3 - 124,505.0)
2024	61,214.0 (59,779.4 - 62,751.5)	62,337.0 (60,513.8 - 64,325.4)	123,551.0 (120,293.2 - 127,076.9)
2025	62,514.7 (61,017.5 - 64,122.4)	63,570.8 (61,673.6 - 65,643.2)	126,085.6 (122,691.1 - 129,765.6)
2026	63,878.2 (62,313.6 - 65,561.8)	64,861.1 (62,885.2 - 67,023.4)	128,739.3 (125,198.8 - 132,585.1)
2027	65,299.1 (63,662.2 - 67,064.7)	66,203.5 (64,144.0 - 68,461.9)	131,502.6 (127,806.3 - 135,526.5)
2028	66,759.1 (65,045.3 - 68,612.7)	67,581.1 (65,433.5 - 69,941.8)	134,340.2 (130,478.8 - 138,554.5)
2029	68,261.5 (66,465.7 - 70,209.7)	68,999.7 (66,758.8 - 71,469.3)	137,261.2 (133,224.5 - 141,679.0)
2030	69,825.1 (67,941.6 - 71,875.5)	70,480.3 (68,140.3 - 73,066.7)	140,305.4 (136,082.0 - 144,942.1)
2031	71,461.2 (69,483.8 - 73,622.2)	72,036.0 (69,590.4 - 74,747.7)	143,497.2 (139,074.2 - 148,369.9)
2032	73,160.4 (71,082.6 - 75,440.7)	73,656.7 (71,099.1 - 76,502.9)	146,817.1 (142,181.8 - 151,943.7)
2033	74,888.7 (72,704.8 - 77,296.8)	75,306.6 (72,631.0 - 78,295.9)	150,195.3 (145,335.9 - 155,592.7)
2034	76,645.1 (74,349.1 - 79,190.1)	76,986.4 (74,186.6 - 80,128.2)	153,631.5 (148,535.7 - 159,318.3)
2035	78,455.3 (76,040.1 - 81,147.6)	78,726.5 (75,795.1 - 82,031.9)	157,181.8 (151,835.3 - 163,179.5)
2036	80,330.5 (77,788.4 - 83,182.0)	80,539.4 (77,468.3 - 84,020.8)	160,869.9 (155,256.7 - 167,202.7)
2037	82,256.3 (79,579.6 - 85,279.4)	82,407.8 (79,188.7 - 86,078.1)	164,664.1 (158,768.2 - 171,357.5)
2038	84,193.4 (81,375.0 - 87,400.6)	84,286.4 (80,912.1 - 88,158.5)	168,479.9 (162,287.1 - 175,559.0)
2039	86,126.0 (83,159.0 - 89,530.0)	86,158.9 (82,622.1 - 90,246.1)	172,284.8 (165,781.1 - 179,776.1)
2040	88,046.4 (84,924.0 - 91,661.3)	88,015.9 (84,309.6 - 92,332.6)	176,062.4 (169,233.6 - 183,993.9)
2041	89,951.7 (86,666.8 - 93,792.5)	89,852.1 (85,969.1 - 94,413.5)	179,803.8 (172,635.9 - 188,206.0)
2042	91,837.9 (88,383.2 - 95,920.8)	91,660.5 (87,593.6 - 96,482.9)	183,498.4 (175,976.9 - 192,403.8)
2043	93,698.1 (90,066.4 - 98,040.8)	93,432.6 (89,174.8 - 98,533.6)	187,130.7 (179,241.3 - 196,574.4)
2044	95,525.6 (91,709.6 - 100,147.5)	95,162.8 (90,706.8 - 100,561.8)	190,688.5 (182,416.5 - 200,709.3)
2045	97,323.8 (93,315.5 - 102,246.4)	96,854.8 (92,192.7 - 102,573.5)	194,178.6 (185,508.2 - 204,819.9)
2046	99,097.3 (94,888.0 - 104,345.1)	98,513.7 (93,636.9 - 104,576.5)	197,611.0 (188,524.9 - 208,921.6)
2047	100,851.5 (96,431.9 - 106,452.0)	100,143.6 (95,042.9 - 106,578.1)	200,995.0 (191,474.8 - 213,030.1)
2048	102,583.3 (97,943.6 - 108,567.2)	101,739.5 (96,405.6 - 108,576.3)	204,322.8 (194,349.1 - 217,143.4)
2049	104,281.3 (99,411.6 - 110,682.3)	103,289.4 (97,712.5 - 110,562.0)	207,570.7 (197,124.1 - 221,244.3)
2050	104,808.5 (99,746.1 - 111,611.5)	103,687.9 (97,907.1 - 111,378.6)	208,496.4 (197,653.1 - 222,990.1)
