# Supplementary material for: Past trends and future projections of palliative care needs in Chile: analysis of routinely available death registry and population data
Source: BMC Med. 2024 Sep 2;22:350. doi: 10.1186/s12916-024-03570-1 (PMC11367822; doi:10.1186/s12916-024-03570-1)
Supplement: Supplementary file 2 — Additional file 2: Methodology supplement. [file 12916_2024_3570_MOESM2_ESM.pdf]

Additional file 2: Methodology supplement.

Method followed to estimate palliative care needs based on the methodology proposed by the Lancet Commission on Global Access to Palliative Care and Pain Relief (LCPCPR)

We estimated the number of descendents and non-descendents with palliative care needs for each year and each of the 20 health conditions proposed by the **Lancet Commission on Global Access to Palliative Care and Pain Relief**. We used the underlying cause of death to identify each person who died from any of the 20 health conditions, year of death, sex and age at death available from the national death registry data between 1997 and 2019.

We used national population forecasts developed by the Institute for National Statistics (INE) to calculated the number of people living in Chile for each year between 1997 to 2050, group of age and sex.

The steps followed for the estimations are described here:

Given a specific condition c $(c = 1, \dots, 20)$,

1) For each condition, we defined as P_{tsg} the total number of deaths in year t, sex s and age group g, $t = 1997, \dots, 2019$, $s = 1\ (males), 2\ (females)$, $g = 20-24, 25-29, \dots, 75-79, \geq 80$.

2) Similarly, we defined as N_{tsg} the total number of people in Chile for year t, sex s and age group g, $t = 1997, \dots, 2050$, $s = 1\ (males), 2\ (females)$, $g = 20-24, 25-29, \dots, 75-79, \geq 80$.

3) We computed a 3-point moving average for the number of deaths by condition c, for year t, $t = 1997, \dots, 2019$, defining

$$P^*_{tsg} = \frac{1}{3}\left(P_{(t-1)sg} + P_{tsg} + P_{(t+1)sg}\right)$$

4) We computed a 3-point moving average for the number of people in Chile for year t, $t = 1997, \dots, 2050$, defining

$$N^*_{tsg} = \frac{1}{3}\left(N_{(t-1)sg} + N_{tsg} + N_{(t+1)sg}\right)$$

For deaths in 1997, 2019, and 2050 a two-point average (current and nearest year) was used. P^*_{tsg} represents a smooth number of deaths for the given condition c, N^*_{tsg} represents a smooth number of people in Chile.

5) For each condition, we fitted a Poisson regression, where the response variable is $P_{tsg}^{*}, t = 1997, \ldots, 2019$, and explanatory variable year, age group and sex. N_{tsg}^{*} was included as the offset.

$$Log(P_{tsg}^{*}) = \beta_0 + \beta_1 \cdot year_t + \beta_2 \cdot sex_s + \beta_3 \cdot agegroup_g + \log(N_{tsg}^{*})$$

6) Using the fitted regression, we predicted the number of deaths for years 2021 to 2050. We will call these $\hat{P}_{tsg}^{*}, t = 2021, \ldots, 2050$.

7) We estimated the number of descendent subjects with palliative care needs for year t, $t = 2021, \ldots, 2050$, sex s, and age group g as the proportion of the total number of deaths for condition c that had declared the Lancet Commission, this is:

$$p_{tsg,c}^{d} = w_c^d \cdot \hat{P}_{tsgc}^{*}$$

where w_c^d denote the Lancet Commission weight for condition c.

8) If the condition had a non-descendent weight defined by the Lancet Commission, then the number of non-descendent subjects with palliative care needs was computed as:

$$p_{tsg,c}^{nd} = w_c^{nd} \cdot \hat{P}_{tsgc}^{*}$$

To obtain the analogous figures for other conditions, we repeated this procedure 20 times (one per condition).

9) To obtain the total number of descendent people who need palliative care each year, we added $p_{tsg,c}^{d}$ across age group, sex, and condition. An analogous procedure was made for non-descendent.
To obtain the total number of people who need palliative care each year, we added $p_{tsg,c}^{d} + p_{tsg,c}^{nd}$ across age group, sex and condition.

Stata scripts

Code 1: Program for predictions

```
* predictions program *

program define predictions

poisson deaths_s i.sexo year i.edad if year<2020, exposure(pop_s) iter(500)
predict post, xb
predict se, stdp

gen ci_lb=post - invnormal(0.975)*se
gen ci_ub=post + invnormal(0.975)*se

gen post_e=exp(post)
gen ci_lb_e=exp(ci_lb)
gen ci_ub_e=exp(ci_ub)

collapse (sum) deaths population deaths_s pop_s post_e ci_lb_e ci_ub_e, by(year)

rename *_e *

twoway (rarea ci_ub ci_lb year, fi(50) col(gs5) lco(white) ) || ///
(line post year, lp(dash) lc(gs5)) || ///
(line deaths_s year if year<2020, lc(red)) ||, ///
name(g`1', replace) ///
title("LCC`1'", size(small)) ///
legend(order(3 "Observed value" 2 "Predicted value" 1 "95% CI") size (*0.6)) ///
graphregion(color(white)) ylabel(, labsize(small)) ///
ytitle("Númer of deaths", size(small) margin(medium)) ///
xlabel(1997(8)2050, labsize(vsmall)) xtitle(" ") nodraw

save "$stata\LCC`1'", replace
end
```

Code 2: Program for palliative care needs from deaths

```
*Program for palliative care needs from deaths*
program drop pallcn
program define pallcn
foreach var of varlist `1' `2' `3' {
gen `var'_PCC=`var'*1 if LCC==1
replace `var'_PCC=`var'*1 if LCC==2
replace `var'_PCC=`var'*1 if LCC==3
replace `var'_PCC=`var'*0.9 if LCC==4
replace `var'_PCC=`var'*0.9 if LCC==5
replace `var'_PCC=`var'*0.8 if LCC==6
replace `var'_PCC=`var'*0.3 if LCC==9
replace `var'_PCC=`var'*0.3 if LCC==10
replace `var'_PCC=`var'*0.3 if LCC==12
```

```
replace `var'_PCC=`var'*0.65 if LCC==13
replace `var'_PCC=`var'*0.5 if LCC==14
replace `var'_PCC=`var'*1 if LCC==15
replace `var'_PCC=`var'*0.65 if LCC==16
replace `var'_PCC=`var'*0.65 if LCC==17
replace `var'_PCC=`var'*0.65 if LCC==18
replace `var'_PCC=`var'*0.7 if LCC==19
replace `var'_PCC=`var'*0.4 if LCC==20
replace `var'_PCC=`var'*0.05 if LCC==22
replace `var'_PCC=`var'*0.8 if LCC==23
replace `var'_PCC=`var'*0.5 if LCC==24
replace `var'_PCC=`var'*0.95 if LCC==25
replace `var'_PCC=`var'*0.45 if LCC==26
replace `var'_PCC=`var'*0.75 if LCC==27
replace `var'_PCC=`var'*0.4 if LCC==28
replace `var'_PCC=`var'*0.6 if LCC==29
replace `var'_PCC=`var'*0.3 if LCC==30
replace `var'_PCC=`var'*0.35 if LCC==31
replace `var'_PCC=`var'*0.7 if LCC==32
replace `var'_PCC=`var'*1 if LCC==33
gen nond`var'_PCC=`var'_PCC*1 if LCC==1
replace nond`var'_PCC=`var'_PCC*0.082 if LCC==2
replace nond`var'_PCC=`var'_PCC*14.87 if LCC==3
replace nond`var'_PCC=`var'_PCC*0.94 if LCC==4
replace nond`var'_PCC=`var'_PCC*3.59 if LCC==6
replace nond`var'_PCC=`var'_PCC*1.67 if LCC==13
replace nond`var'_PCC=`var'_PCC*1.67 if LCC==14
replace nond`var'_PCC=`var'_PCC*1.67 if LCC==15
replace nond`var'_PCC=`var'_PCC*1.67 if LCC==16
replace nond`var'_PCC=`var'_PCC*0.95 if LCC==17
replace nond`var'_PCC=`var'_PCC*1 if LCC==29
replace nond`var'_PCC=`var'_PCC*1.9996 if LCC==30
replace nond`var'_PCC=`var'_PCC*2 if LCC==32
replace nond`var'_PCC=0 if nond`var'_PCC==.
}
End
```

Code 3: Population data from INE

```
import delimited "$def\Poblacion.txt", delimiter("\t") varn(1)
keep edad sexo y1997-y2050

replace edad="100" if edad=="100+"
destring edad, replace
gen edad2=0 if edad==0
replace edad2=1 if edad>0 & edad<5
replace edad2=2 if edad>=5 & edad<10
replace edad2=3 if edad>=10 & edad<15
replace edad2=4 if edad>=15 & edad<20
replace edad2=5 if edad>=20 & edad<25
```

```
replace edad2=6 if edad>=25 & edad<30
replace edad2=7 if edad>=30 & edad<35
replace edad2=8 if edad>=35 & edad<40
replace edad2=9 if edad>=40 & edad<45
replace edad2=10 if edad>=45 & edad<50
replace edad2=11 if edad>=50 & edad<55
replace edad2=12 if edad>=55 & edad<60
replace edad2=13 if edad>=60 & edad<65
replace edad2=14 if edad>=65 & edad<70
replace edad2=15 if edad>=70 & edad<75
replace edad2=16 if edad>=75 & edad<80
replace edad2=17 if edad>=80 & edad<85
replace edad2=18 if edad>=85
drop edad
rename edad2 edad
rename y* p*

collapse (sum) p*, by(edad sexo)

save "$data\population.dta", replace
```

Code 4: Mortality data from DEIS

```
import delimited "$stata\DEFUNCIONES_FUENTE_DEIS_1990_2020-2.csv", delim(";")
varn(nonames)

tab agecode, miss

gen edad=age if agecode=="1"
replace edad=0 if agecode=="2" | agecode=="3" | agecode=="4"
tab edad agecode

gen edad2=0 if edad==0
replace edad2=1 if edad>0 & edad<5
replace edad2=2 if edad>=5 & edad<10
replace edad2=3 if edad>=10 & edad<15
replace edad2=4 if edad>=15 & edad<20
replace edad2=5 if edad>=20 & edad<25
replace edad2=6 if edad>=25 & edad<30
replace edad2=7 if edad>=30 & edad<35
replace edad2=8 if edad>=35 & edad<40
replace edad2=9 if edad>=40 & edad<45
replace edad2=10 if edad>=45 & edad<50
replace edad2=11 if edad>=50 & edad<55
replace edad2=12 if edad>=55 & edad<60
replace edad2=13 if edad>=60 & edad<65
replace edad2=14 if edad>=65 & edad<70
replace edad2=15 if edad>=70 & edad<75
replace edad2=16 if edad>=75 & edad<80
replace edad2=17 if edad>=80 & edad<85
```

```
replace edad2=18 if edad>=85
drop edad agecode age
rename edad2 edad

label define edadlab 0"<1" 1"1-4" 2"5-9" 3"10-14" 4"15-19" 5"20-24" ///
6"25-29" 7"30-34" 8"35-39" 9"40-44" 10"45-49" 11"50-54" 12"55-59" ///
13"60-64" 14"65-69" 15"70-74" 16"75-79" 17"80-84" 18">84", replace
label values edad edadlab

gen sexo=1 if sex=="Hombre"
replace sexo=2 if sex=="Mujer"
drop if sexo==.
drop if yod<=1996

gen deaths=1
collapse (sum) deaths, by(yod sexo causa1 edad)


*Keeping causes of death from Lancet Commissioning codes*
rename causa1 cie10

gen LCC=1 if cie10=="B334"
replace LCC=2 if cie10>="A150" & cie10<"A20"
replace LCC=3 if cie10>="B200" & cie10<"B250"
replace LCC=4 if cie10>="C000" & cie10<"C91"
replace LCC=4 if cie10>="C960" & cie10<"C980"
replace LCC=5 if cie10>="C910" & cie10<"C960"
replace LCC=6 if cie10>="F000" & cie10<="F040"
replace LCC=6 if cie10>="G300" & cie10<"G330"

replace LCC=7 if cie10>="G01" & cie10<"G02"
replace LCC=8 if cie10>="G02" & cie10<"G03"
replace LCC=9 if cie10>="G00" & cie10<"G01"
replace LCC=9 if cie10>="G03" & cie10<"G04"
replace LCC=10 if cie10>="G040" & cie10<"G06"
replace LCC=11 if cie10=="G028"
replace LCC=12 if cie10>="G06" & cie10<"G10"

replace LCC=13 if cie10>="G200" & cie10<"G270"
replace LCC=14 if cie10>="G400" & cie10<"G420"
replace LCC=15 if cie10>="G350" & cie10<"G380"
replace LCC=16 if cie10>="G800" & cie10<"G840"

replace LCC=17 if cie10>="I600" & cie10<="I70"

replace LCC=18 if cie10>="I050" & cie10<"I100"
replace LCC=19 if cie10>="I100" & cie10<"I160"
replace LCC=20 if cie10>="I420" & cie10<"I430"
replace LCC=20 if cie10>="I500" & cie10<"I60"

replace LCC=22 if cie10>="I250" & cie10<"I26"
```

```
replace LCC=23 if cie10>="J400" & cie10<"J48"
replace LCC=24 if cie10>="J600" & cie10<"J71"
replace LCC=24 if cie10>="J800" & cie10<"J85"
replace LCC=24 if cie10>="J950" & cie10<="J999"

replace LCC=25 if cie10>="K700" & cie10<="K770"
replace LCC=26 if cie10>="N170" & cie10<"N20"

replace LCC=27 if cie10>="P070" & cie10<"P08"
replace LCC=28 if cie10>="P100" & cie10<"P160"

replace LCC=29 if cie10>="Q000" & cie10<="Q999"
replace LCC=30 if cie10>="S000" & cie10<="S999"
replace LCC=30 if cie10>="T000" & cie10<="T989"
replace LCC=30 if cie10>="V010" & cie10<="Y989"
replace LCC=31 if cie10>="I700" & cie10<"I71"
replace LCC=32 if cie10>="M000" & cie10<="M979"
replace LCC=33 if cie10>="E400" & cie10<"E47"
tab LCC, miss

drop if LCC==.
drop cie10
tab yod

save "$data\Deaths_1997-20.dta", replace
```

Code 5: Analysis

```
use "$data\Deaths_1997-20.dta", clear
rename yod year

collapse (sum) deaths, by(year edad sexo LCC)

reshape wide deaths , i(edad sexo LCC) j(year )
rename deaths* d*
reshape long d, i(edad sexo LCC) j(year)

reshape wide d, i(edad LCC year) j(sexo)
reshape long d, i(edad LCC year) j(sexo)
reshape wide d, i(edad sexo year) j(LCC)
reshape long d, i(edad sexo year) j(LCC)

replace d=0 if d==.

reshape wide d, i(edad sexo LCC) j(year)

forvalues i=2021(1)2050{
   gen d`i'=.
}
```

```
merge m:1 edad sexo using "$data\population.dta"
drop _merge

reshape long d p, i(sexo edad LCC) j(year)

rename d deaths
rename p population

sort  LCC  sexo edad year
replace deaths=. if year==2020


gen deaths_s=(deaths + deaths[_n-1] + deaths[_n+1])/3
replace deaths_s=(deaths + deaths[_n+1])/2 if year==1997
replace deaths_s=(deaths + deaths[_n-1])/2 if year==2019

gen pop_s=(population + population[_n-1] + population[_n+1])/3
replace pop_s=(population + population[_n+1])/2 if year==1997
replace pop_s=(population + population[_n-1])/2 if year==2050

collapse (sum) deaths population deaths_s pop_s , by(LCC year sexo edad )
replace deaths=. if year>2019
replace deaths_s=. if year>2019

order year LCC sexo edad
sort year LCC sexo edad

tab LCC

save "$stata\for_poisson92-50.dta", replace

foreach i of numlist 1 2 3 4 5 6 9 10 12 13 14 15 16 17 18 19 20 22 23 24 25 26 27 28 29 30 31 32
33 {
use "$stata\for_poisson92-50.dta", clear
keep if LCC==`i'
predictions `i'
}

grc1leg g1 g2 g3 g4 g5 g6 g9 g10 g12 g13 g14 g15 g16 g17 g18 g19 g20  g22 g23 g24 g25 g26 g27
g28 g29 g30 g31 g32 g33 , graphregion(color(white)) iscale(*0.75)

use "$stata\LCC1", clear
gen LCC=1

foreach i of numlist  2 3 4 5 6 9 10 12 13 14 15 16 17 18 19 20 22 23 24 25 26 27 28 29 30 31 32 33
{
	append  using "$stata\LCC`i'"
	replace LCC=`i' if LCC==.
}
```

```
tab LCC, miss

pallcn post ci_lb ci_ub


pallcn deaths_s

rename post* predicted*
rename nond* ND*
rename *_s_* *_*
rename *ci_ub* *UCI*
rename *ci_lb* *LCI*

save "$stata\data for graphs.dta", replace
```
